# Genomic and transcriptomic analyses of thyroid cancers identify DICER1 somatic mutations in adult follicular-patterned RAS-like tumors

**DOI:** 10.3389/fendo.2023.1267499

**Published:** 2023-10-05

**Authors:** Emanuela Minna, Andrea Devecchi, Federico Pistore, Biagio Paolini, Giuseppe Mauro, Donata Alda Penso, Sonia Pagliardini, Adele Busico, Giancarlo Pruneri, Loris De Cecco, Maria Grazia Borrello, Marialuisa Sensi, Angela Greco

**Affiliations:** ^1^ Pathology Unit 2, Department of Diagnostic Innovation, Fondazione IRCCS Istituto Nazionale dei Tumori, Milan, Italy; ^2^ Integrated Biology of Rare Tumors, Department of Experimental Oncology, Fondazione IRCCS Istituto Nazionale dei Tumori, Milan, Italy; ^3^ Pathology Unit 1, Fondazione IRCCS Istituto Nazionale dei Tumori, Milan, Italy; ^4^ Department of Oncology and Hemato-Oncology, University of Milan, Milan, Italy; ^5^ Department of Diagnostic Innovation, Fondazione IRCCS Istituto Nazionale dei Tumori, Milan, Italy; ^6^ Platform of Integrated Biology, Department of Applied Research and Technology Development, Fondazione IRCCS Istituto Nazionale dei Tumori, Milan, Italy

**Keywords:** thyroid cancer, whole exome sequencing, transcriptomics, mutations, *DICER1*

## Abstract

**Background:**

Papillary thyroid carcinoma (PTC) is the most common type of thyroid cancer (TC). Several genomic and transcriptomic studies explored the molecular landscape of follicular cell-derived TCs, and *BRAF*V600E, *RAS* mutations, and gene fusions are well-established drivers. *DICER1* mutations were described in specific sets of TC patients but represent a rare event in adult TC patients.

**Methods:**

Here, we report the molecular characterization of 30 retrospective follicular cell-derived thyroid tumors, comprising PTCs (90%) and poorly differentiated TCs (10%), collected at our Institute. We performed DNA whole-exome sequencing using patient-matched control for somatic mutation calling, and targeted RNA-seq for gene fusion detection. Transcriptional profiles established in the same cohort by microarray were investigated using three signaling-related gene signatures derived from The Cancer Genome Atlas (TCGA).

**Results:**

The occurrence of *BRAF*V600E (44%), *RAS* mutations (13%), and gene fusions (13%) was confirmed in our cohort. In addition, in two patients lacking known drivers, mutations of the *DICER1* gene (p.D1709N and p.D1810V) were identified. *DICER1* mutations occur in two adult patients with follicular-pattern lesions, and in one of them a second concurrent *DICER1* mutation (p.R459*) is also observed. Additional putative drivers include *ROS1* gene (p.P2130A mutation), identified in a patient with a rare solid-trabecular subtype of PTC. Transcriptomics indicates that *DICER1* tumors are RAS-like, whereas the *ROS1*-mutated tumor displays a borderline RAS-/BRAF-like subtype. We also provide an overview of *DICER1* and *ROS1* mutations in thyroid lesions by investigating the COSMIC database.

**Conclusion:**

Even though small, our series recapitulates the genetic background of PTC. Furthermore, we identified *DICER1* mutations, one of which is previously unreported in thyroid lesions. For these less common alterations and for patients with unknown drivers, we provide signaling information applying TCGA-derived classification.

## Introduction

Follicular cell-derived tumors represent the majority of thyroid cancers (TCs) and encompass various histological types and subtypes. Based on histological features, they are classified as well-differentiated tumors, comprising papillary thyroid carcinoma (PTC) and follicular thyroid carcinoma (FTC), and poorly differentiated and undifferentiated thyroid carcinomas (PDTCs and ATCs, respectively). It is recognized that these less-differentiated tumors can develop from preexisting PTC or FTC according to a model of sequential dedifferentiation process and accumulation of multiple genetic abnormalities (1, 2).

PTC is the most common type in both adult and pediatric thyroid malignancies (3) and represents a heterogeneous disease with several subtypes that differ in terms of histological and clinical features, as well as molecular alterations. The most frequent and studied subtypes are the classical, follicular, and tall cells (4, 5), whereas other subtypes with solid and/or trabecular growth patterns exist (6) but are less characterized due to their rarity.

PTC and FTC display distinctive characteristics; PTC (especially the classical subtype) displays papillary architecture, specific nuclear morphological changes, and preferential metastatic dissemination *via* lymphatic vessels. FTC instead displays follicular architecture and can retain thyroid cell differentiation but lacks the PTC nuclear features and metastasizes preferentially *via* blood vessels (7). Thyroid lesions can be thus papillary- or follicular-patterned based on tumor origin and on these features. Follicular-patterned lesions include benign, low-risk, and malignant neoplasms, such as the follicular adenoma (FA), the PTC follicular subtype, FTC, FTC-derived PDTC, and other less common entities (8).

Along with the histopathological classification, molecular studies have then demonstrated that specific genetic alterations occur in given TC types, driving carcinogenesis according to a genotype/phenotype correlation (1, 4). In well-differentiated TCs in general, a very low mutational burden is observed compared with other cancers (9) and few somatic mutations or mutually exclusive gene fusions involving effectors of the mitogen-activated protein kinase (MAPK) and phosphatidylinositol 3-kinase (PI3K) signaling cascades are identified. In PTC, the *BRAF*V600E mutation is the most common genetic alteration followed by *RET* and *NTRK1/3* tyrosine kinase receptor gene fusions (4, 10). These drivers are particularly enriched in the PTC tall cell and classical subtypes, whereas somatic mutations of *RAS* gene family members *NRAS*, *HRAS*, and *KRAS* (mostly affected at codon 61, and less often at codon 12/13) are more frequent in the PTC follicular subtype and in FTC (5, 11, 12). The same alterations can be found in PDTC and ATC along with additional mutations in PI3K-AKT pathway genes and other well-established cancer-associated genes (such as *TP53*, *TERT* promoter, chromatin remodeling, and DNA damage response genes (12, 13)), in agreement with the sequential accumulation of gene alterations promoting tumor progression.

In addition to the well-known gene drivers, in more recent years with the advancement of sequencing technologies, several other genes have emerged as altered in TC. For instance, mutations in the *DICER1* gene, coding for an RNase III endoribonuclease involved in microRNA biogenesis, were identified as a rare event in adult TCs (5, 11–16), whereas they were more frequently reported in pediatric TC patients (8, 17–20) and in carriers of the DICER1 syndrome (21–27), an inherited cancer-predisposing disorder caused by germline *DICER1* mutations. DICER1 syndrome patients display a wide spectrum of neoplasias with early onset, including thyroid nodular goiter, follicular adenoma, and differentiated TC (28). The occurrence of thyroid manifestations, as multi-nodular goiter in children and young adults and in a familial context, has been even proposed as an early event to identify DICER1 syndrome families (29, 30). In the lesions of DICER1 syndrome patients, the co-occurrence of a germline *DICER1* variant, often loss-of-function, with a second missense somatic mutation was observed, and tumorigenesis induced by double-hit mutations has been proposed (31).

Subsequent TC omics studies have demonstrated that the identified mutations in the MAPK pathway stimulate specific transcriptional programs, affecting the downstream extracellular signal–regulated kinases (ERK) signaling, the expression of thyroid differentiation and function genes, and the activation of proliferative and immune-inflammatory programs. In particular, based on the expression of gene signatures, The Cancer Genome Atlas (TCGA) consortium defined in PTC three transcriptional signatures, related to the presence of *BRAF*V600E *vs. RAS* mutations, to the degree of retained thyroid differentiation and to MAPK pathway output (5). These transcriptional signatures have been subsequently validated in various TC types and cohorts (11, 13, 32–34), also from our laboratory (35–37). It is now established indeed that *BRAF*V600E- and *RAS*-mutated PTCs display a signaling defined BRAF-like and RAS-like, respectively, and that tumors with other drivers can display BRAF-like, RAS-like, or intermediate/borderline signaling. Tumors with *RET/PTC1*, for instance, are BRAF-like, whereas other gene fusions can be RAS-like (5, 11). PDTCs are frequently RAS-like, even though BRAF-like subtypes can be also identified (13). Similarly, different transcriptional subtypes referred to thyroid differentiation (TD) and to MAPK pathway output have been established, with *BRAF*V600E and BRAF-like tumor displaying loss of TD and higher activation of the MAPK pathway, which could explain the worse prognosis in *BRAF*V600E- compared with *RAS*-mutated patients (4).

In this study, we profiled by DNA whole-exome sequencing, targeted RNA-sequencing, and transcriptomics 30 follicular cell-derived thyroid tumors, comprising both PTCs and PDTCs, collected at our Institute, to classify them according to known and novel genomic driver alterations and to TCGA-defined thyroid cancer-related transcriptional subtypes.

## Materials and methods

### Caselist collection

A retrospective caselist of thyroid cancer patients collected at our Institute was selected based on (i) confirmed diagnosis of follicular cell-derived thyroid tumor and (ii) availability of residual archive material from both tumor and matched non-neoplastic thyroid (NT), included as patient-specific control for DNA sequencing. The obtained cohort included both various PTC histological subtypes and PDTCs. PTCs were classified according to the WHO classification of endocrine tumors (38) and PDTCs according to the Turin proposal (39). Formalin-fixed paraffin-embedded (FFPE) tissue blocks were obtained, and hematoxylin and eosin (H&E)-stained slides were reviewed by an experienced pathologist (PB); when necessary, the areas of interest were manually microdissected. Primary tumor and patient-matched NT were obtained from 57 patients and subjected to nucleic acid extraction and quality control, for a total of 126 processed samples.

The study was approved by the Independent Ethics Committee of Fondazione IRCCS Istituto Nazionale dei Tumori (protocol INT DI-20/12/13-0006020), and informed consent was obtained from patients.

### Nucleic acid extraction

Nucleic acids were extracted from unstained FFPE tissue serial sections consecutive to the pathologically revised H&E. Genomic DNA and total RNA were extracted by GeneRead DNA FFPE kit and by miRNeasy FFPE kit (Qiagen, Hilden, Germany), respectively, using the QIAcube-automated purification system. Extracted nucleic acids were quantified by Qubit 4 Fluorometer using Qubit Assay Kits (Thermo Fisher Scientific, Waltham, MA, USA), and quality was assessed by TapeStation 4200 (Agilent Technologies, Santa Clara, CA, USA) using Agilent ScreenTape Assays. Only patients with adequate DNA quantity (total extracted DNA >200 ng) from both tumor and matched NT were processed for DNA sequencing.

### Whole exome sequencing and data processing

Tumor/NT pairs derived from 32 patients underwent library construction. DNA was fragmented by a Covaris M220 sonicator, and libraries were prepared using Illumina TruSeq Exome Library Prep Kit (Illumina, San Diego, CA, USA) according to the manufacturer’s instructions. A total of 60 samples, corresponding to 30 patient-matched tumor/NT pairs, passed library quality control on TapeStation 4200 (Agilent Technologies) and were submitted to library pooling and sequencing on Illumina NextSeq500 System according to the manufacturer’s standard protocol.

DNA sequencing data were processed as previously reported (40). Briefly, raw fastq files were quality-controlled with FastQC (41) and paired-end reads were aligned to the reference human genome (hg19) using a Burrows–Wheeler Aligner (BWAMEM, v0.7.12) (42). Duplicate and unmapped reads were identified and removed with Picard software (http://broadinstitute.github.io/picard/) and SAMtools v1.3.1.31. Reads were then post-processed according to Genome Analysis Toolkit (GATK) Best Practices 3.7 which include left alignment of small insertions and deletions, indel realignment, and base quality score recalibration.

Somatic single-nucleotide variants (SNVs) and small indels were called by two different variant callers: MuTect2 (v3.7) and Strelka (v.29.10). To create a high-confidence variant list, only the variants called by both algorithms were considered. Somatic variants (substitutions and indels) were annotated with Oncotator (v1.9.9.0). To remove false positives and polymorphisms, variants were then excluded based on at least one of the following additional filters: (i) read depth <30 in both tumor and NT; (ii) unidirectional call; (iii) alternative allele present in matched NT if the variant was not listed in the Catalogue Of Somatic Mutations In Cancer (COSMIC) database; (iv) C>A/G>T variants with a frequency <0.1 (oxoG artifacts); and (v) variants annotated in polymorphism databases (ExAC (43), NCBI dbSNP (44), and 1000 Genome Project (45)) without a COSMIC annotation. COSMIC Cancer Gene Census (https://cancer.sanger.ac.uk/census) was interrogated to explore the impact of somatic mutations in selected genes.

### Targeted RNA sequencing for gene fusion detection

Gene fusions were assessed on total RNA by Oncomine™ Comprehensive Assay Plus RNA panel (OCA Plus RNA, Thermo Fisher Scientific) that covers more than 1,300 isoforms across 49 known cancer-related fusion drivers. Libraries were prepared using the Ion AmpliSeq Library Kit plus with OCA RNA plus pools and sequenced on an Ion GeneStudio S5 Prime sequencer using Ion 530 chips, Ion 510 & Ion 520 & Ion 530 Kit-Chef, and Ion Chef System (Thermo Fisher Scientific), according to the manufacturer’s instructions. Data were processed by Torrent Suite™ and analyzed by Ion Reporter™ software (5.18 version) with the “Oncomine Comprehensive Plus - w2.2 - Fusions - Single Sample” workflow.

### Transcriptomics

Gene expression profiles were established by microarrays using Clariom S Pico Assay (Thermo Fisher Scientific). Total RNA was reverse transcribed, amplified, fragmented, biotin-labeled, and hybridized to Affymetrix GeneChip Human Clariom S (CLS) Arrays according to the manufacturer’s standard protocols. Washing and staining procedures were performed using the GeneChip Hybridization, Wash and Stain Kit (Thermo Fisher Scientific) on Affymetrix GeneChip Fluidics Station 450. Microarrays were scanned with the GeneChip Scanner 3000 7G system (Thermo Fisher Scientific), and data were obtained using Affymetrix GeneChip Command Console (AGCC) software.

Data were processed using the robust multi-array average (RMA) algorithm on paraffin samples (46); raw Affymetrix CEL file data were background-noise-adjusted, normalized, and log2-transformed using the oligo package and RMA function. Probes were annotated with Bioconductor annotation package clariomshumantranscriptcluster.db, whereas probes not associated with gene symbols and control probes were filtered out. Multiple probes mapping to the same gene were collapsed using the collapseRows function with the “MaxMean” method of the WGCNA package (47). All analyses were performed using RStudio version 4.0.3. Microarray data were deposited in the ArrayExpress repository with the accession number E-MTAB-13222.

Transcriptional subtypes were defined using three gene signatures derived from TCGA (5); the complete gene lists are published (5, 33), and the corresponding expression scores were calculated as previously reported (37). Briefly, the BRAF-/RAS-like signaling gene set comprises 71 genes; 69 out of 71 genes were available on the used CLS array and assessed for score computation. The BRAF-RAS score (BRS) was calculated as reported (48). Negative BRS values were defined as BRAF-like subtype, whereas positive BRS values were defined as RAS-like subtype as reported (5); close-to-zero BRS values were considered as borderline subtype. Thyroid differentiation (TD) and MAPK output gene sets comprise 16 and 52 genes, respectively. TD and MAPK output scores were calculated as mean of log2-transformed and median-centered expression across samples as previously reported (5, 33). Positive and negative TD score values were defined as high and low expressions of thyroid function genes, respectively. Positive and negative MAPK output score values were defined high and low MAPK pathway transcriptional activation, respectively.

### Meta-analysis of DICER1 and ROS1 mutations in thyroid tissues from COSMIC

The COSMIC database (https://cancer.sanger.ac.uk/cosmic, accessed on 30 January 2023) was interrogated to explore *DICER1* (COSMIC gene COSG526495; transcript ENST00000526495.5) and *ROS1* (COSMIC gene COSG418; transcript ENST00000368508.7) gene mutations. Thyroid tissue was specifically investigated, and the linked data for each gene were downloaded.

*DICER1* and *ROS1* mutations were reported in 24 and 8 different studies, respectively. We focused on the 19 and 6 studies reporting at least one mutated sample, respectively. For *DICER1*, an additional study derived from literature (11), and not included in COSMIC, was also considered.

Data derived from published studies were checked on the original publication to confirm mutation type, tissue histology, and patient-matched samples. Data derived from CGP (Cancer Genome Project) studies were manually curated: (i) samples from CGP study_542 were assigned to TCGA study (5) based on ID matching; (ii) one sample from CGP study_542 (COSMIC ID 2122053) with *ROS1* synonymous mutation was excluded; and (iii) duplicated samples from CGP study_676 were excluded.

## Results

### Caselist description

**Figure 1:**
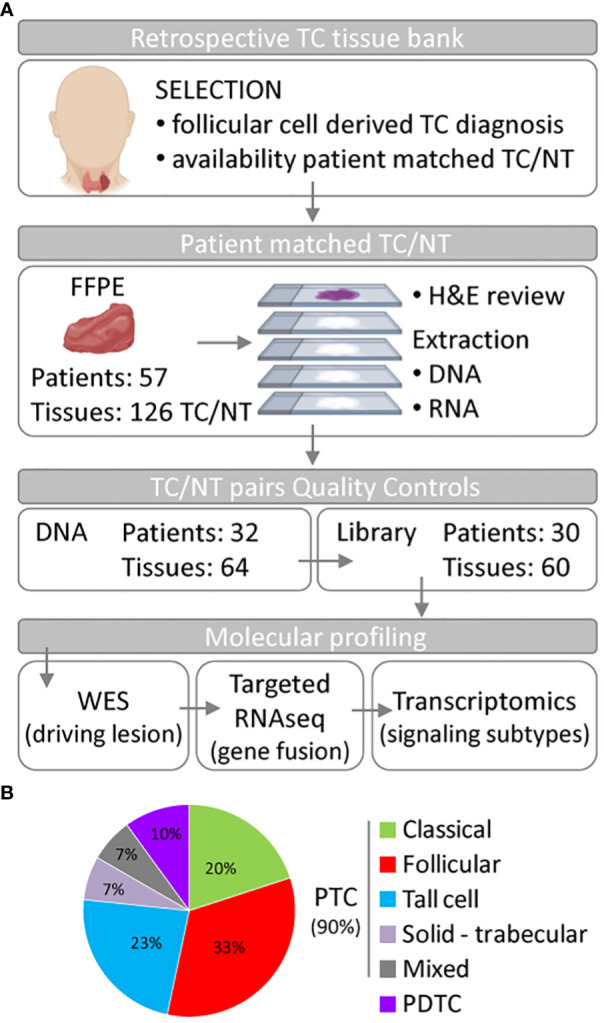
Summary of the thyroid cancer caselist processing and features. **(A)** Flowchart of thyroid tissue selection and processing to obtain the set of 30 thyroid cancer (TC) patients who undergone genomic and transcriptomic molecular profiling. Part of this Figure has been created using Biorender under institutional account. **(B)** Pie chart of the histological subtype distribution in the 30 TC patients. Abbreviations: NT, non-neoplastic thyroid; FFPE, formalin-fixed paraffin-embedded tissue; H&E, hematoxylin and eosin; WES, whole-exome sequencing; PTC, papillary thyroid carcinoma; PDTC, poorly differentiated thyroid carcinoma.

We investigated a retrospective series of TC patients collected at Fondazione IRCCS Istituto Nazionale dei Tumori of Milan. Caselist selection and processing are described in Material and Methods, **Figure 1A** and **Supplementary Figure 1**. Starting from 126 FFPE tissues derived from 57 patients, and comprising TCs and patient-matched non-neoplastic thyroids (NTs), a final set of 30 tumor/NT pairs (collectively 60 tissues) passed quality control standards and were profiled first by DNA whole-exome sequencing (WES) and then by targeted RNA sequencing and transcriptomics (**Figure 1A**). Clinicopathological features are reported in **Table 1** and **Supplementary Table 1**. This series includes PTCs (90%) and PDTCs (10%). PTC histological types comprise classical and follicular subtypes, the less frequent tall cell and solid-trabecular subtypes, and two cases with mixed components (**Figure 1B**, **Table 1** and **Supplementary Table 1**), thus being representative of the histological heterogeneity observed in this tumor type.

**Table 1:** Feature of the 30 TC patients.

Gender: female/male		24/6
Age (years): median (Range)		42 (13–74)
Tumor size (cm): median (range)		2 (1.2-6.5)
ETE	Yes; n (%)	17 (57%)
	No; n (%)	11 (37%)
	NA; n (%)	2 (7%)
LNM	Yes; n (%)	11 (37%)
	No; n (%)	19 (63%)
Histological subtype; n (%)		
PTC	Classical	6 (20%)
	Follicular	10 (33%)
	Tall cell	7 (23%)
	Solid—trabecular	2 (7%)
	Mixed	2 (7%)
PDTC		3 (10%)

ETE, extra thyroid extension; LNM, lymph node metastases; PTC; papillary thyroid carcinoma; PDTC poorly differentiated thyroid carcinoma; NA, not available.

### Somatic mutations

DNA WES was established using patient-matched NT as filtering control for somatic mutation calling. We specifically focused on non-synonymous mutations, causing amino acid (aa) changing, and comprising missense, nonsense, and splice-site mutations with aa changing. The identified mutations for each tumor are in **Supplementary Table 2**.

**Figure 2:**
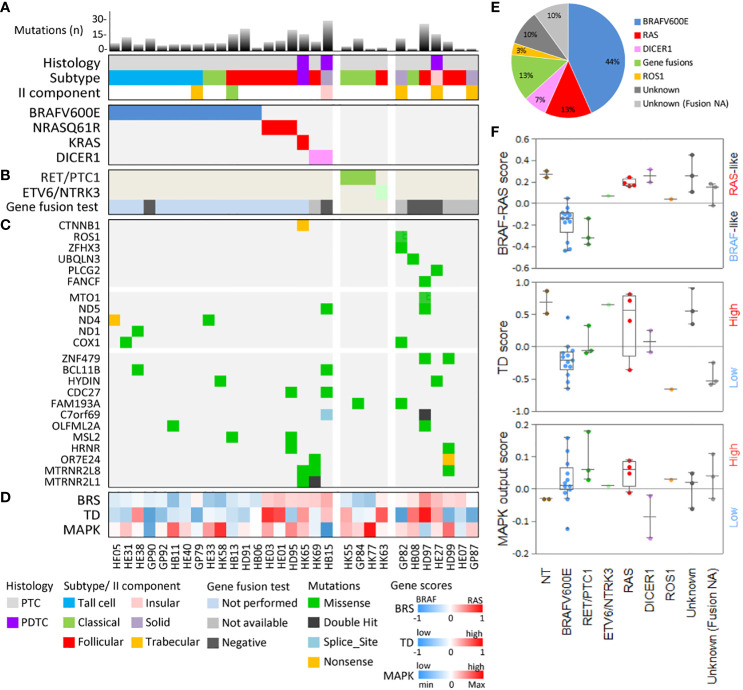
Genomic and transcriptomic characterization of the 30 thyroid cancer patients. **(A)** The mutational load (bar chart, top panel), histology features (middle panel), and driver/putative driver mutations (lower panel) are shown for each patient. Mutational load represents the number of somatic non-synonymous mutations (missense, nonsense, and splice-site mutations with amino acid changing). **(B)** Gene fusion detected by targeted RNA-seq. **(C)** Selected genes mutated in our cohort, including mitochondrial function-related genes (middle panel) and commonly mutated genes (lower panel). **(D)** Signaling subtypes defined by gene expression score based on TCGA gene signatures related to BRAF-RAS signaling (BRAF-/RAS-like score, BRS), thyroid differentiation (TD score), and activation status of the MAPK pathway (MAPK output score). Patient identifiers and color-code legends are shown at the bottom. **(E)** Pie chart showing the distribution of driver/putative driver alterations in the 30 TC patients. **(F)** Boxplot with scatter plot showing signaling subtypes in the 30 TC patients stratified for alteration. Two non-neoplastic thyroids (NTs) are included as controls. Tumors with unknown driver and gene fusion data not available (NA) are shown as a separate group.

The mutation load of our set (**Figure 2A** and **Supplementary Figure 2**) was low in agreement with that of thyroid cancers from TCGA (**Supplementary Figure 2A**) and from other TC series (5, 12, 13). The median number of mutations was 6.5 (range 1–28), with the majority of samples (19/30, 63%) harboring less than 10 mutations (**Supplementary Figure 2B**). The top mutated samples (number of mutations ≥15) included all the three tumors with PDTC component/histology (**Supplementary Figure 2B**), in agreement with the higher mutational burden reported in advanced and less differentiated TCs (12, 13). A significant correlation between patient age and mutation load was also observed (**Supplementary Figure 2C**), confirming a lower mutation load in younger patients (**Supplementary Figure 2D**) as previously reported (12).

#### Driver/putative driver alterations

The most frequently altered genes were related to the MAPK pathway and included well-established gene drivers (**Figures 2A, E**). *BRAF*V600E was the most frequent mutation (13/30 samples, 44%), followed by *RAS* mutations (4/30 samples, 13%), with *NRAS*_Q61R identified in three patients and *KRAS*_G12V, co-occurring with a beta-catenin (*CTNNB1*) mutation, in another patient.

In samples lacking the abovementioned alterations (**Figure 2A**), mutations in the *DICER1* gene were detected. A meta-analysis of *DICER1* mutation in TC is presented hereafter.

The identified mutations were mutually exclusive (**Figure 2A**) and displayed a genotype/histological subtype distribution (**Supplementary Table 1**), as already observed in other TC series. Most *BRAF*V600E were found in PTC classical and tall cell subtypes (10/13, 77%), whereas *RAS* mutations were found in the PTC follicular subtype (3/4, 75%) and in a PDTC with the PTC component where *KRAS* and *CTNNB1* mutations were co-occurring. *DICER1* mutations were found in a follicular subtype PTC and in a PDTC with solid-insular histology. This agrees with the increasing body of evidence describing *DICER1* mutations in TCs with a follicular pattern rather than a papillary pattern (49).

Collectively, we identified these somatic mutations in known/putative drivers in 19/30 cases (64%) (**Figure 2A**).

#### Meta-analysis of DICER1 mutations in TC

**Figure 3:**
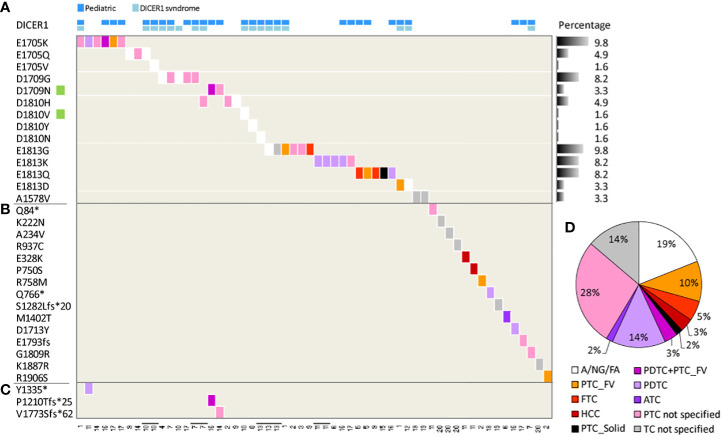
*DICER1* mutations in thyroid lesions derived from COSMIC. Heatmap of *DICER1* mutations in 53 patients across 20 studies. The tissue histology is shown, when available, according to the color-code legend on the right; the study identifier (see **Table 2**) is at the bottom. Each column represents a patient; four patients with multiple tissues tested are displayed as adjacent lanes, highlighted by bolt lines on study identifier. *DICER1* mutations (row) are listed based on **(A)** most frequently mutated codons, the percentage across samples (n = 61) reported; **(B)** patient-specific mutations; **(C)** second-hit somatic mutations. Green squares on the mutation list highlight the two mutations identified also in the present study. **(D)** Pie chart of histological subtypes distribution; all the specimens (n = 58) were considered. Abbreviation: A, adenoma; NG, nodular goiter, FA, follicular adenoma; TC, thyroid carcinoma; PTC, papillary TC; FV, follicular variant/subtype; FTC, follicular TC; HCC, hurtle cell carcinoma; solid, solid subtype; PDTC, poorly differentiated TC; ATC, anaplastic TC. A/NG/FA were computed as a single class of benign lesions. PTC and TC non-specified classes include samples with a non-specified histological subtype.

We used as primary information source the COSMIC database. Mutations in *DICER1* are reported by 20 independent studies (collectively 1669 samples, **Table 2**), describing 61 *DICER1* mutations (3.6%) in 53 patients. It should be noted that for some patients multiple specimens were tested (**Table 2** and **Figure 3**) and that some pedigrees of DICER1 syndrome carriers are included, thus possibly representing a slight overestimation of *DICER1* mutation frequency in TC. Specific hotspots are frequently identified and include the functionally relevant codons E1705, D1709, D1810, and E1813, all representing metal ion-binding sites localized within the DICER1 RNase IIIb domain and affecting its enzymatic activity (21). In our cohort, *DICER1* alterations (i.e., D1709N VAF 0.94 and D1810V VAF 0.37, highlighted in **Figure 3**) are found in two of these hotspots, thus falling into the same functional category, and display amino acid substitutions previously reported (12, 19, 25).

**Table 2:** COSMIC-derived thyroid studies reporting *DICER1* mutation.

	Study ID	Mutations (n)	Patients (n)	Total reported samples (n)	Reference
1	de Kock _JCEM2014	3	3	3	(21)
2	TCGA_Cell2014*	4	4	402	(5)
3	Costa_Oncotarget2015	1	1	18	(14)
4	de Kock _JTO2016	1	1	1	(22)
5	Durieux_VirchArchiv2016	2	2	2	(17)
6	Landa_JCI2016	2	2	117	(13)
7	Rutter _JCEM2016	4^1^	3	5	(23)
8	Wu_ERC2016	1	1	1	(24)
9	Yoo_PlosGen2016 **	4	4	180	(11)
10	Apellaniz-Ruiz_EJE2017	4^1^	3	6	(25)
11	Zehir_NatMed2017	7^1,2^	5	233	(15)
12	Chen_JCO2018	1	1	1	(26)
13	Gullo_AJCP2018	3^1^	1	5	(27)
14	Pozdeyev_CCR2018	4^2^	3	631	(12)
15	Ravella_AnnPathologie2018	1	1	1	(18)
16	Chernock _ModernPath2020	6^2^	5	7	(19)
17	Lee_JCI2021	5	5	37	(20)
18	Kim_InVivo2022	1	1	12	(16)
19	CGP Study_589	3	3	3	NA
20	CGP Study_676*	4	4	4	NA
	Total	61	53	1669	

* Manually curated (see Material and methods). ** Not included in COSMIC-derived studies.

^1^Multiple samples from the same patient. ^2^ DICER1 double mutation. NA, not available.

In addition, in HK69 patient, *DICER1*_D1810V co-occurs with *DICER1*_R459* mutation (VAF 0.43, splice-site mutations with stop codon introduction; **Supplementary Table 2**), localized into the DICER1 helicase C domain. To our knowledge, this mutation has not been previously reported in other thyroid patients but is listed in the *DICER1* mutation panel (50). Notably, a frame-shift loss-of-function mutation at the N458 residue concurring with *DICER1*_D1810Y, both somatic, has been recently reported in an adult TC patient (51). In addition, co-occurring *DICER1* mutations have been similarly identified in TC patients not related to DICER1 syndrome (28, 51–53) and also in COSMIC patients (**Figure 3C**) where RNase IIIb domain mutations (at codons E1705 and D1709) co-occur with a second nonsense/frameshift_nonsense mutation.

Focusing on the histology (**Figure 3D**), our meta-analysis confirms that *DICER1* mutations can frequently occur in follicular-pattern lesions, as PTC follicular subtype (either alone or with the PDTC component), PTC solid subtype, FTC, and Hurtle cell carcinoma (collectively 20%), as well as in benign/premalignant lesions (adenoma, nodular goiter, and follicular adenoma, collectively computed as unique class, 19%), and also in less differentiated TCs (PDTC+ATC 19%).

### Gene fusions

As reported in the previous section, 11/30 cases did not display mutations in the abovementioned known/putative drivers. We therefore investigated these samples for the presence of gene fusions, well-established drivers in PTC, by a targeted RNA sequencing panel covering 49 cancer-related chromosomal rearrangements (see Material and Methods). In the analysis, we also included *DICER1*-mutated samples, as the co-occurrence of a *DICER1* mutation with a rare gene fusion has been reported (14);and one *BRAF*V600E sample, included as negative control.

*RET* and *NTRK3* fusions were found in four cases, all lacking mutations in *BRAF*, *RAS*, and *DICER1* genes, thus confirming to be mutually exclusive with each other and with other known/putative drivers (**Figure 2B**). The identified fusions were *RET/PTC1* (fusion partner *CCDC6* gene) in three cases and *ETV6/NTRK3* in one case, both representing the oncogenic fusions more frequently identified in PTC. *RET/PTC1* fusions were found in the PTC classical subtype, whereas *ETV6/NTRK3* was found in the follicular subtype; this agrees with the genotype/histological subtype distribution observed in other TC series (5, 11) and with the described association of *NTRK* fusions with the follicular growth pattern (54). Negative samples were one *DICER1*-mutated sample, the negative control (*BRAF*V600E), and three samples, which thus remained with the unknown gene driver. Five samples did not pass assay quality control due to low-quality RNA, thus displaying not available data for this analysis.

Collectively, considering also gene fusions, we identified gene alterations in 23/30 samples (77%), whereas 7 samples remained with unknown driver (**Figure 2E**).

### Samples with unknown drivers

To identify additional potential drivers in our cohort, we then revised somatic mutation data (**Supplementary Table 2**).

In one patient, a *ROS1* gene mutation was detected (**Figure 2C**). *ROS1* codes for a receptor tyrosine kinase and is included in Cosmic Cancer Gene Census as containing mutations causally implicated in cancer. While its rearrangement has been found in various tumor types (55), including recent TC case reports (56, 57), its mutation seems a quite rare event in thyroid tumors. In the COSMIC database, 25 *ROS1* mutations (2%) are described across 1,215 samples (**Supplementary Table 3**). No common mutations are reported, and the *ROS1*_P2130A mutation detected in our patient (VAF 0.18, **Supplementary Table 2**) represents a new alteration for this gene in thyroid lesions. Other drivers (as *BRAF*V600E or *HRAS*, **Supplementary Table 3**) co-occur with *ROS1* mutations, thus raising the possibility that this gene may not represent a standalone driver in TC. However, in our patient, mutations in known drivers are absent, thus suggesting that other alterations may be involved; due to unavailable data, we cannot confirm the absence of gene fusion (see previous section). Interestingly, in our patient *ROS1* mutation co-occurs with *ZFHX3*_A472E (**Supplementary Table 2**), and the same co-occurrence is observed in COSMIC (**Supplementary Table 3**; COSMIC ID 2121935). The meaning and impact of these data remain to be established. Notably, *ZFHX3* mutations co-occur with other gene drivers in 1.7% of PTC from TCGA (5).

In our cohort, the *ROS1* mutation was found in a PTC with the rare solid-trabecular histology, whereas the few available COSMIC data (**Supplementary Table 3**) describe *ROS1* mutations in both follicular-pattern tumors (as FTC and FTA), as well as in ATC and *BRAF*V600E PTCs, thus suggesting that the current data are sparse and insufficient to address a genotype/phenotype association for *ROS1* mutations in TC.

Revising the other samples with unknown drivers, especially the three with confirmed absence of gene fusions (i.e. sample ID HB08, HD97, HE27; **Figures 2B, C**), we found a variable number of somatic mutations (range 1–25, **Figure 2A**); mostly were missense mutations (**Supplementary Table 2**) and affected genes previously reported in other cancer types, such as *UBQLN3*, *PLCG2*, and *FANCF* genes (**Figure 2C**).

In addition, in the HD97 patient, mutations in two genes encoding mitochondrial proteins were identified: the mitochondrial gene *ND5* (NADH dehydrogenase subunit 5, p.F429L mutation) and the nuclear gene *MTO1* (mitochondrial tRNA translation optimization 1, p.M386L mutation) (**Figure 2C** and **Supplementary Table 2**). Of note, mutations in other mitochondrial genes were identified in six other patients of our caselist, and affecting *ND4* and *ND1* genes (NADH dehydrogenase subunit 4 and subunit 1), involved in the activity of the mitochondrial membrane NADH dehydrogenase (complex I), which catalyzes the electron transfer from NADH through the respiratory chain, and the *COX1* gene (cytochrome c oxidase I) involved in the electron transport in mitochondrial respiratory chain complex III and IV (**Figure 2C**). Mutations in mitochondrial genes have been previously identified in thyroid cancer. Nonsense disruptive mitochondrial DNA mutations in complex I subunits have been reported in the oncocytic variant of thyroid carcinoma (58), and more recently in other TC subtypes (59–61). Interestingly, the *ND4* gene mutation here identified (*ND4*_W24*, **Supplementary Table 2**) is nonsense, possibly affecting complex I formation. Furthermore, four of the patients with mitochondrial gene mutations are *BRAF*V600E positive, in agreement with a previously suggested correlation between *BRAF* mutation and mitochondrial alterations in TC (62).

Along with mitochondrial genes, other genes were found commonly mutated in both samples with unknown and known/putative driver (**Figure 2C**). These genes belong to different functional categories and cellular processes, such as cell proliferation (*CDC27* gene), ERK signaling (*HYDIN* gene), transcription regulation by DNA binding (*BCL11B* and *ZNF479* genes), extracellular matrix organization (*OLFML2A* gene), chromatin organization (*MSL2* gene), calcium ion binding (*HRNR* gene), and apoptosis regulation (*MTRNR2L8* and *MTRNR2L1*), or are less characterized genes (**Figure 2C** and **Supplementary Table 2**).

To get more information about the pathways affected by the identified mutated genes, we performed a pathway-level analysis (**Supplementary Figure 3**). The somatic mutations found in our cohort (**Supplementary Table 2**) were tested on the Hallmark collection derived from Molecular Signature Database (63). We found that several mutated genes fall into biological processes related to (i) cell proliferation; (ii) p53 and apoptosis; (iii) stress responses as hypoxia and DNA damage; (iv) signaling pathways associated with KRAS, MTOR, TNFA, and estrogen receptor; (v) metabolic functions; and (vi) inflammation, consistently with the biological alterations and features typically observed in cancer (64). The vast majority of the genes identified in these pathways, however, are altered in samples with a known/putative driver, whereas very limited information is obtained for the unknown samples.

### Transcriptional subtypes

In our cohort, we then established transcriptional profiles by RNA microarray; two normal thyroids were included as control. We tested our samples with the three TCGA-derived gene signatures related to BRAF/RAS signaling (BRAF-/RAS-like subtypes), thyroid differentiation (TD score), and activation status of the MAPK pathway (MAPK output) (5). This was aimed not only to validate these transcriptional subtypes in samples with recognized drivers, but also, and more importantly, to obtain signaling information for the samples with putative and unknown drivers.

For NT controls and samples with established drivers, we confirmed literature findings. Normal thyroids displayed, as expected, a high TD score (5), low MAPK output, and RAS-like subtype as previously reported (34, 36) (**Figure 2F**). *BRAF*V600E samples were BRAF-like, with a low TD score and high MAPK output (4) (**Figures 2D, F**). Samples with *RET/PTC1* fusion showed results similar to *BRAF*V600E samples, except for the higher TD score (**Figure 2F**) indicative of a partial preservation of thyroid function, as already reported (5, 35). On the contrary, *RAS*-mutated samples were confirmed as RAS-like, with higher TD score and intermediate MAPK output. Also, *DICER1*-mutated samples were RAS-like, consistently with previous reports (5, 14), and with the histological subtypes (follicular and PDTC solid-insular) in which these alterations were found. Of note, they displayed on average TD scores lower than those of *RAS* and higher than those of *BRAF*V600E samples, and reduced MAPK output, consistently with previous data (65). The *ETV6/NTRK3* sample displayed a borderline RAS-/BRAF-like signaling subtypes, as already reported (5, 11).

Regarding the samples with unknown driver, the one with *ROS1* mutation displayed a borderline RAS-/BRAF-like subtype, whereas the remaining samples were RAS-like, consistently with their histology (mostly follicular and solid/insular/trabecular) and displayed intermediate MAPK output and a heterogeneous degree of thyroid differentiation.

## Discussion

In this study, we report the molecular characterization of 30 TCs collected at our Institute. We applied DNA WES on tumor/normal thyroid patient-matched tissues, targeted RNA sequencing for gene fusion testing in samples negative for known driving mutations, and transcriptomics to assess TCGA-derived signaling subtypes.

Even though small, our cohort includes both the most frequent and less common histological subtypes of PTC, as well as a minor fraction of PDTCs, thus being representative of various histological types observed in follicular cell-derived thyroid tumors.

We confirmed literature data about the low mutational burden in well-differentiated TC and younger patients, the occurrence and distribution of well-established gene drivers, and their genotype/phenotype association.

In samples lacking *BRAF*V600E, *RAS* mutations, and *RET* and *NTRK* gene fusions, we found mutations in the *DICER1* gene. Alterations in this gene have been identified by various independent studies, as also highlighted by our COSMIC meta-analysis, raising the possibility of its involvement in TC tumorigenesis.

We described three different *DICER1* mutations; the two affecting the functionally relevant and frequently altered hotspots in the RNase IIIb domain have been already reported in thyroid lesions, whereas the *DICER1*_R459* has been previously identified only in pleuropulmonary blastoma (66). Mutations of *DICER1* in thyroid tumors are quite rare (3.6% from COSMIC). Notably, in our small cohort, we detected a higher alteration frequency (two mutation-positive patients out of 30, 6.6%). This increased detection could be explained by the composition of our cohort, comprising a high fraction of follicular-pattern/RAS-like tumors.

Although numerous studies have identified *DICER1* mutations in thyroid lesions, particularly in pediatric TC patients and DICER1 syndrome carriers, the functional meaning of these alterations still remains poorly understood. DICER1 operates in mature miRNA synthesis, and recently it has been confirmed that actually thyroid lesions with *DICER1* mutations in the RNase IIIb domain display an altered mature miRNA transcriptome compared with *DICER1* wild-type tumors and non-neoplastic thyroids (67, 68). Unfortunately, we are not able to test mature miRNA expression in transcriptomic data from our cohort as the exploited microarray platform is not designed for short RNA assessment, and mature miRNA data are not available.

In our cohort, somatic *DICER1* mutations were identified in two adult patients: a follicular subtype PTC (with missense *DICER1*_D1810V and nonsense *DICER1*_R459*) and a PDTC with solid-insular histology (with missense *DICER1*_D1709N), respectively, in agreement with the enrichment of follicular-pattern TCs observed in *DICER1*-mutated patients (49). Other more recent studies, not included in the COSMIC-derived dataset, have reported *DICER1* mutations in thyroid neoplasms. They confirmed not only hotspot mutations in the *DICER1* RNAse IIIb domain, including the D1810V and D1709N mutations identified in our samples, but also the co-occurrence of second loss-of-function mutations (28, 51–53), as well as the enrichment of *DICER1* mutations in follicular pattern thyroid tumors. A revision of these studies is available at reference (28).

Given the increasing number of studies reporting *DICER1* mutations, this gene has been recognized among relevant molecular markers for TC (6) and its testing has been included in thyroid-specific NGS panels, such as the gene test ThyroSeq v3 (69). In addition, as previously reported, in the presence of two somatic *DICER1* mutations in the same tumor tissue, germline *DICER1* testing should be taken into account to confirm the not inherited nature of the case, and to exclude a DICER1 syndrome-related manifestation for which specific management, surveillance strategies, and follow-up have been recommended (70).

In one sample with an unknown driver, we identified the *ROS1*_P2130A mutation. To our knowledge, this alteration has not been previously reported in thyroid lesions but has been detected in a lung adenocarcinoma patient (71). Although this mutation affects a conserved amino acid in the kinase domain of the protein, its potential role as genetic driver in thyroid cancer remains to be established.

Interestingly, the *ROS1*-mutated sample displayed a borderline RAS-/BRAF-like subtype that could be explained by the co-occurrence of other drivers (including gene fusions, undetermined in this sample) that may affect the transcriptional signaling. Indeed, PTCs carrying both *ROS1* and *BRAF*V600E mutations are BRAF-like (5), whereas an ATC with co-occurring *ROS1* and PI3K pathway mutations is RAS-like (13). Further studies are required to define the impact and role of *ROS1* mutations in TC.

In the other samples with an unknown driver, we identified missense mutations in *UBQLN3*, *PLCG2*, and *FANCF*, among other genes (**Supplementary Table 2**).

The mutation in the *UBQLN3* gene (*UBQLN3*_R624Q), encoding a ubiquitin-like protein, is detected in a classical subtype PTC (HB08 patient). Of note, this represents the only somatic mutation identified in this patient. UBQLN3 belongs to the ubiquilins protein family, essential factors for the maintenance of cell proteostasis and found involved in cancer progression. *UBQLN3* missense mutations have been reported in lung, breast, central nervous system, and pancreatic cancer, although their functional role in cancer remains unexplored (72).

The mutation in *PLCG2* genes (*PLCG2*_R653C), encoding the Phospholipase Cgamma 2 enzyme, is identified in a follicular subtype PTC (HE27 patient). *PLCG2* missense and nonsense mutations are reported in 2% of all cancers (https://www.mycancergenome.org/content/gene/plcg2) as in colon cancer, lung cancer, prostate cancer, endometrial carcinoma, and cutaneous melanoma, as well as in ibrutinib-resistant chronic lymphocytic leukemia patients (73). Of note, a *PLCG2* mutation of unknown significance has been found in an ATC with *BRAF*V600E mutation (74).

The mutation in the *FANCF* gene (*FANCF*_A81V), encoding the DNA repair protein Fanconi Anemia Complementation group F, is identified in a PDTC (HD97 patient). FANCF is an adaptor protein of the Fanconi Anemia core complex and plays a key role in DNA post-replication repair and in cell-cycle checkpoints. Mutations in *FANCF* are frequently observed in human tumors as breast, lung, kidney, and ovary (75). Moreover, *FANCF* promoter methylation has been found in cancer and a *FANCF*-deficient mouse model was prone to ovarian cancers (76). The mutation of other elements of the Fanconi Anemia core complex and of genes involved in DNA damage response has been already observed in TC, especially in advanced and less differentiated tumors (12, 13). The significance of the here identified mutation remains to be investigated.

In the same patient (HD97), we also identified missense mutations in mitochondrial genes. Multiple mutations of genes related to mitochondrial function were found in our cohort, in agreement with the body of evidence showing aberrant mitochondrial function in cancer.

In addition, we found several genes commonly mutated across our samples; they are still poorly characterized both at the functional level and for a possible involvement in cancer, and further studies are mandatory to assess the impact of the here identified mutations.

To decipher the possible pathways and biological processes affected by the mutations identified in our cohort, we performed a pathway-level analysis. We found that several of the identified mutations converge on relevant biological processes already recognized as altered in cancer. However, the vast majority of the genes identified in these pathways were altered in samples with known/putative drivers, whereas very limited information was obtained for the unknown samples, which still remain largely uncharacterized. In this sense, the availability of matched transcriptomic data for these patients may be further explored in future to dissect downstream changes in gene expression and obtain more information about the altered functions and pathways in driver unknown patients.

In conclusion, here we described genomic and transcriptomic data for a proprietary cohort of thyroid cancer patients. Even though small, our cohort, mostly consisting of PTC, recapitulates the well-established genetic background for this tumor type. Moreover, in adult patients with follicular-pattern tumors, we described *DICER1* mutations, one of which is previously unreported in TC. In addition, our study suggested several putative driver alterations, including a ROS1 mutation, whose role in TC remains to be investigated. We also provided signaling subtype information applying the well-established TCGA-derived classification, thus unveiling the molecular features of TCs carrying less common and poorly characterized gene mutations.

## Data availability statement

The gene expression data presented in this study can be found in the online repository ArrayExpress (https://www.ebi.ac.uk/biostudies/arrayexpress) with the accession number E-MTAB-13222. The raw WES data presented in the study are not publicly available since they contain information that could compromise research participant privacy. Pre-processed somatic mutation data are included in the article/**Supplementary Material (Supplementary Table 2)**. Further inquiries can be directed to the corresponding author/s.

## Ethics statement

The studies involving humans were approved by Independent Ethics Committee of Fondazione IRCCS Istituto Nazionale dei Tumori (protocol INT DI-20/12/13-0006020). The studies were conducted in accordance with the local legislation and institutional requirements. The participants provided their written informed consent to participate in this study.

## Author contributions

EM: Data curation, Formal Analysis, Investigation, Validation, Visualization, Writing – original draft, Writing – review & editing. AD: Data curation, Formal Analysis, Methodology, Software, Validation, Writing – review & editing. FP: Data curation, Formal Analysis, Software, Validation, Writing – review & editing. BP: Validation, Writing – review & editing, Investigation. GM: Data curation, Investigation, Writing – review & editing. DAP: Investigation, Validation, Writing – review & editing. SP: Investigation, Writing – review & editing. AB: Formal Analysis, Investigation, Validation, Writing – review & editing. GP: Resources, Writing – review & editing. LDC: Methodology, Supervision, Writing – review & editing. MGB: Conceptualization, Investigation, Writing – review & editing. MS: Conceptualization, Formal Analysis, Supervision, Writing – review & editing. AG: Conceptualization, Funding acquisition, Project administration, Resources, Supervision, Writing – review & editing.

## References

[1] Xing M. Molecular pathogenesis and mechanisms of thyroid cancer. Nat Rev Cancer (2013) 13:184–99. doi: 10.1038/nrc3431 PMC3791171 23429735

[2] Cabanillas ME McFadden DG Durante C. Thyroid cancer. Lancet (2016) 388:2783–95. doi: 10.1016/S0140-6736(16)30172-6 27240885

[3] Kitahara CM Sosa JA. Understanding the ever-changing incidence of thyroid cancer. Nat Rev Endocrinol (2020) 16:617–8. doi: 10.1038/s41574-020-00414-9 PMC7476643 32895503

[4] Fagin JA Wells SA. Biologic and clinical perspectives on thyroid cancer. N Engl J Med (2016) 375:1054–67. doi: 10.1056/NEJMra1501993 PMC5512163 27626519

[5] Agrawal N Akbani R Aksoy BA Ally A Arachchi H Asa SL. Integrated genomic characterization of papillary thyroid carcinoma. Cell (2014) 159:676–90. doi: 10.1016/j.cell.2014.09.050 PMC4243044 25417114

[6] Baloch ZW Asa SL Barletta JA Ghossein RA Juhlin CC Jung CK. Overview of the 2022 WHO classification of thyroid neoplasms. Endocr Pathol (2022) 33:27–63. doi: 10.1007/s12022-022-09707-3 35288841

[7] Haugen BR Alexander EK Bible KC Doherty GM Mandel SJ Nikiforov YE. 2015 American thyroid association management guidelines for adult patients with thyroid nodules and differentiated thyroid cancer: the american thyroid association guidelines task force on thyroid nodules and differentiated thyroid cancer. Thyroid (2016) 26:1–133. doi: 10.1089/thy.2015.0020 26462967 PMC4739132

[8] Bae JS Jung SH Hirokawa M Bychkov A Miyauchi A Lee S. High prevalence of DICER1 mutations and low frequency of gene fusions in pediatric follicular-patterned tumors of the thyroid. Endocr Pathol (2021) 32:336–46. doi: 10.1007/s12022-021-09688-9 34313965

[9] Lawrence MS Stojanov P Polak P Kryukov GV Cibulskis K Sivachenko A. Mutational heterogeneity in cancer and the search for new cancer-associated genes. Nature (2013) 499:214–8. doi: 10.1038/nature12213 PMC3919509 23770567

[10] Kondo T Ezzat S Asa SL. Pathogenetic mechanisms in thyroid follicular-cell neoplasia. Nat Rev Cancer (2006) 6:292–306. doi: 10.1038/nrc1836 16557281

[11] Yoo SK Lee S Kim Sj Jee HG Kim BA Cho H. Comprehensive analysis of the transcriptional and mutational landscape of follicular and papillary thyroid cancers. PloS Genet (2016) 12:e1006239. doi: 10.1371/journal.pgen.1006239 27494611 PMC4975456

[12] Pozdeyev N Gay LM Sokol ES Hartmaier R Deaver KE Davis S. Genetic analysis of 779 advanced differentiated and anaplastic thyroid cancers. Clin Cancer Res (2018) 24:3059–68. doi: 10.1158/1078-0432.CCR-18-0373 PMC6030480 29615459

[13] Landa I Ibrahimpasic T Boucai L Sinha R Knauf JA Shah RH. Genomic and transcriptomic hallmarks of poorly differentiated and anaplastic thyroid cancers. J Clin Invest (2016) 126:1052–66. doi: 10.1172/JCI85271 PMC4767360 26878173

[14] Costa V Esposito R Ziviello C Sepe R Bim LV Cacciola NA. New somatic mutations and WNK1-B4GALNT3 gene fusion in papillary thyroid carcinoma. Oncotarget (2015) 6:11242–51. doi: 10.18632/oncotarget.3593 PMC4484453 25803323

[15] Zehir A Benayed R Shah RH Syed A Middha S Kim HR. Mutational landscape of metastatic cancer revealed from prospective clinical sequencing of 10,000 patients. Nat Med (2017) 23:703–13. doi: 10.1038/nm.4333 PMC5461196 28481359

[16] Kim JH Jeong JY Seo AN Park NJY Kim M Park JY. Genomic profiling of aggressive thyroid cancer in association with its clinicopathological characteristics. In Vivo (2022) 36:111–20. doi: 10.21873/invivo.12682 PMC8765130 34972706

[17] Durieux E Descotes F Mauduit C Decaussin M Guyetant S Devouassoux-Shisheboran M. The co-occurrence of an ovarian Sertoli-Leydig cell tumor with a thyroid carcinoma is highly suggestive of a DICER1 syndrome. Virchows Arch Int J Pathol (2016) 468:631–6. doi: 10.1007/s00428-016-1922-0 26983701

[18] Ravella L Lopez J Descotes F Lifante JC David C Decaussin-Petrucci M. [DICER1 mutated, solid/trabecular thyroid papillary carcinoma in an 11-year-old child]. Ann Pathol (2018) 38:316–20. doi: 10.1016/j.annpat.2018.04.003 29884466

[19] Chernock RD Rivera B Borrelli N Hill DA Fahiminiya S Shah T. Poorly differentiated thyroid carcinoma of childhood and adolescence: a distinct entity characterized by DICER1 mutations. Mod Pathol (2020) 33:1264–74. doi: 10.1038/s41379-020-0458-7 PMC7329587 31937902

[20] Lee YA Lee H Im SW Song YS Oh DY Kang HJ. *NTRK* and *RET* fusion–directed therapy in pediatric thyroid cancer yields a tumor response and radioiodine uptake. J Clin Invest (2021) 131:e144847. doi: 10.1172/JCI144847 34237031 PMC8439610

[21] de Kock L Sabbaghian N Soglio DBD Guillerman RP Park BK Chami R. Exploring the association Between DICER1 mutations and differentiated thyroid carcinoma. J Clin Endocrinol Metab (2014) 99:E1072–1077. doi: 10.1210/jc.2013-4206 24617712

[22] de Kock L Bah I Wu Y Xie M Priest JR Foulkes WD. Germline and somatic DICER1 mutations in a well-differentiated fetal adenocarcinoma of the lung. J Thorac Oncol (2016) 11:e31–33. doi: 10.1016/j.jtho.2015.09.012 26886166

[23] Rutter MM Jha P Schultz KAP Sheil A Harris AK Bauer AJ. DICER1 mutations and differentiated thyroid carcinoma: evidence of a direct association. J Clin Endocrinol Metab (2016) 101:1–5. doi: 10.1210/jc.2015-2169 26555935 PMC4701837

[24] Wu MK de Kock L Conwell LS Stewart CJR King BR Choong CS. Functional characterization of multiple DICER1 mutations in an adolescent. Endocr Relat Cancer (2016) 23:L1–5. doi: 10.1530/ERC-15-0460 26545620

[25] Apellaniz-Ruiz M de Kock L Sabbaghian N Guaraldi F Ghizzoni L Beccuti G. Familial multinodular goiter and Sertoli-Leydig cell tumors associated with a large intragenic in-frame DICER1 deletion. Eur J Endocrinol (2018) 178:K11–9. doi: 10.1530/EJE-17-0904 29187512

[26] Chen KS Stuart SH Stroup EK Shukla AS Wang J Rajaram V. Distinct DICER1 hotspot mutations identify bilateral tumors as separate events. JCO Precis Oncol (2018) 2. doi: 10.1200/PO.17.00113 PMC6938390 31893257

[27] Gullo I Batista R Rodrigues-Pereira P Soares P Barroca H do Bom-Sucesso M. Multinodular goiter progression toward Malignancy in a case of DICER1 syndrome: histologic and molecular alterations. Am J Clin Pathol (2018) 149:379–86. doi: 10.1093/ajcp/aqy004 29538609

[28] Ghossein CA Dogan S Farhat N Landa I Xu B. Expanding the spectrum of thyroid carcinoma with somatic DICER1 mutation: a survey of 829 thyroid carcinomas using MSK-IMPACT next generation sequencing platform. Virchows Arch Int J Pathol (2022) 480:293–302. doi: 10.1007/s00428-021-03212-4 PMC10126990 34580763

[29] Khan NE Bauer AJ Schultz KAP Doros L Decastro RM Ling A. Quantification of thyroid cancer and multinodular goiter risk in the DICER1 syndrome: A family-based cohort study. J Clin Endocrinol Metab (2017) 102:1614–22. doi: 10.1210/jc.2016-2954 PMC5443331 28323992

[30] Oliver-Petit I Bertozzi AI Grunenwald S Gambart M Pigeon-Kerchiche P Sadoul JL. Multinodular goitre is a gateway for molecular testing of DICER1 syndrome. Clin Endocrinol (2019) 91:669–75. doi: 10.1111/cen.14074 31408196

[31] Thunders M Delahunt B. Gene of the month: DICER1: ruler and controller. J Clin Pathol (2021) 74:69–72. doi: 10.1136/jclinpath-2020-207203 33293352

[32] Yoo SK Song YS Lee EK Hwang J Kim HH Jung G. Integrative analysis of genomic and transcriptomic characteristics associated with progression of aggressive thyroid cancer. Nat Commun (2019) 10:2764. doi: 10.1038/s41467-019-10680-5 31235699 PMC6591357

[33] Dunn LA Sherman EJ Baxi SS Tchekmedyian V Grewal RK Larson SM. Vemurafenib redifferentiation of BRAF mutant, RAI-refractory thyroid cancers. J Clin Endocrinol Metab (2018) 104:1417–28. doi: 10.1210/jc.2018-01478 PMC6435099 30256977

[34] Kim K Jeon S Kim TM Jung CK. Immune gene signature delineates a subclass of papillary thyroid cancer with unfavorable clinical outcomes. Cancers (2018) 10:494. doi: 10.3390/cancers10120494 30563160 PMC6316581

[35] Colombo C Minna E Gargiuli C Muzza M Dugo M De Cecco L. The molecular and gene/miRNA expression profiles of radioiodine resistant papillary thyroid cancer. J Exp Clin Cancer Res (2020) 39:245. doi: 10.1186/s13046-020-01757-x 33198784 PMC7667839

[36] Minna E Brich S Todoerti K Pilotti S Collini P Bonaldi E. Cancer associated fibroblasts and senescent thyroid cells in the invasive front of thyroid carcinoma. Cancers (2020) 12:112. doi: 10.3390/cancers12010112 31906302 PMC7016563

[37] Bonaldi E Gargiuli C De Cecco L Micali A Rizzetti MG Greco A. BRAF inhibitors induce feedback activation of RAS pathway in thyroid cancer cells. Int J Mol Sci (2021) 22:5744. doi: 10.3390/ijms22115744 34072194 PMC8198461

[38] Lloyd RV Osamura RY Klöppel G Rosai J. WHO classification of tumours of endocrine organs. In: WHO Clasification of Tumors, 4th edition. Lyon, France: International Agency for Research on Cancer (IARC (2017). p. 355.

[39] Volante M Collini P Nikiforov YE Sakamoto A Kakudo K Katoh R. Poorly differentiated thyroid carcinoma: the Turin proposal for the use of uniform diagnostic criteria and an algorithmic diagnostic approach. Am J Surg Pathol (2007) 31:1256–64. doi: 10.1097/PAS.0b013e3180309e6a 17667551

[40] Ziccheddu B Biancon G Bagnoli F De Philippis C Maura F Rustad EH. Integrative analysis of the genomic and transcriptomic landscape of double-refractory multiple myeloma. Blood Adv (2020) 4:830–44. doi: 10.1182/bloodadvances.2019000779 PMC7065476 32126144

[41] Babraham Bioinformatics - FastQC A Quality Control tool for High Throughput Sequence Data. Available at: https://www.bioinformatics.babraham.ac.uk/projects/fastqc/.

[42] Li H Durbin R. Fast and accurate long-read alignment with Burrows-Wheeler transform. Bioinforma Oxf Engl (2010) 26:589–95. doi: 10.1093/bioinformatics/btp698 PMC2828108 20080505

[43] Lek M Karczewski KJ Minikel EV Samocha KE Banks E Fennell T. Analysis of protein-coding genetic variation in 60,706 humans. Nature (2016) 536:285–91. doi: 10.1038/nature19057 PMC5018207 27535533

[44] Sherry ST Ward MH Kholodov M Baker J Phan L Smigielski EM. dbSNP: the NCBI database of genetic variation. Nucleic Acids Res (2001) 29:308–11. doi: 10.1093/nar/29.1.308 PMC29783 11125122

[45] 1000 Genomes Project Consortium Abecasis GR Auton A Brooks LD DePristo MA Durbin RM. An integrated map of genetic variation from 1,092 human genomes. Nature (2012) 491:56–65. doi: 10.1038/nature11632 23128226 PMC3498066

[46] Irizarry RA Hobbs B Collin F Beazer-Barclay YD Antonellis KJ Scherf U. Exploration, normalization, and summaries of high density oligonucleotide array probe level data. Biostat Oxf Engl (2003) 4:249–64. doi: 10.1093/biostatistics/4.2.249 12925520

[47] Zhang B Horvath S. A general framework for weighted gene co-expression network analysis. Stat Appl Genet Mol Biol (2005) 4. doi: 10.2202/1544-6115.1128 16646834

[48] Rusinek D Pfeifer A Cieslicka M Kowalska M Pawlaczek A Krajewska J. TERT promoter mutations and their impact on gene expression profile in papillary thyroid carcinoma. Cancers (2020) 12:1597. doi: 10.3390/cancers12061597 32560331 PMC7352936

[49] Sauer M Barletta JA. Proceedings of the north american society of head and neck pathology, los angeles, CA, March 20, 2022: DICER1-related thyroid tumors. Head Neck Pathol (2022) 16:190–9. doi: 10.1007/s12105-022-01417-w PMC9018915 35307774

[50] Hata A Kashima R. Dysregulation of microRNA biogenesis machinery in cancer. Crit Rev Biochem Mol Biol (2016) 51:121–34. doi: 10.3109/10409238.2015.1117054 PMC5226641 26628006

[51] Chong AS Nikiforov YE Condello V Wald AI Nikiforova MN Foulkes WD. Prevalence and spectrum of DICER1 mutations in adult-onset thyroid nodules with indeterminate cytology. J Clin Endocrinol Metab (2021) 106:968–77. doi: 10.1210/clinem/dgab025 33460435

[52] Wasserman JD Sabbaghian N Fahiminiya S Chami R Mete O Acker M. DICER1 mutations are frequent in adolescent-onset papillary thyroid carcinoma. J Clin Endocrinol Metab (2018) 103:2009–15. doi: 10.1210/jc.2017-02698 29474644

[53] Lee YA Im SW Jung KC Chung EJ Shin CH Kim JI. Predominant DICER1 pathogenic variants in pediatric follicular thyroid carcinomas. Thyroid (2020) 30:1120–31. doi: 10.1089/thy.2019.0233 32228164

[54] Pekova B Sykorova V Mastnikova K Vaclavikova E Moravcova J Vlcek P. NTRK fusion genes in thyroid carcinomas: clinicopathological characteristics and their impacts on prognosis. Cancers (2021) 13:1932. doi: 10.3390/cancers13081932 33923728 PMC8073383

[55] Drilon A Jenkins C Iyer S Schoenfeld A Keddy C Davare MA. ROS1-dependent cancers — biology, diagnostics and therapeutics. Nat Rev Clin Oncol (2021) 18:35–55. doi: 10.1038/s41571-020-0408-9 32760015 PMC8830365

[56] Ritterhouse LL Wirth LJ Randolph GW Sadow PM Ross DS Liddy W. ROS1 rearrangement in thyroid cancer. Thyroid (2016) 26:794–7. doi: 10.1089/thy.2016.0101 27089969

[57] Liu SV Macke LA Colton BS Imran SS Christiansen J Chow-Maneval E. Response to entrectinib in differentiated thyroid cancer with a ROS1 fusion. JCO Precis Oncol (2017) 1. doi: 10.1200/PO.17.00105 PMC7446518 32913977

[58] Gasparre G Porcelli AM Bonora E Pennisi LF Toller M Iommarini L. Disruptive mitochondrial DNA mutations in complex I subunits are markers of oncocytic phenotype in thyroid tumors. Proc Natl Acad Sci USA (2007) 104:9001–6. doi: 10.1073/pnas.0703056104 PMC1885617 17517629

[59] Gopal RK Kübler K Calvo SE Polak P Livitz D Rosebrock D. Widespread chromosomal losses and mitochondrial DNA alterations as genetic drivers in hürthle cell carcinoma. Cancer Cell (2018) 34:242–255.e5. doi: 10.1016/j.ccell.2018.06.013 30107175 PMC6121811

[60] Ganly I Makarov V Deraje S Dong Y Reznik E Seshan V. Integrated genomic analysis of hürthle cell cancer reveals oncogenic drivers, recurrent mitochondrial mutations, and unique chromosomal landscapes. Cancer Cell (2018) 34:256–270.e5. doi: 10.1016/j.ccell.2018.07.002 30107176 PMC6247912

[61] Dabravolski SA Nikiforov NG Zhuravlev AD Orekhov NA Mikhaleva LM Orekhov AN. The role of altered mitochondrial metabolism in thyroid cancer development and mitochondria-targeted thyroid cancer treatment. Int J Mol Sci (2021) 23:460. doi: 10.3390/ijms23010460 35008887 PMC8745127

[62] Tsybrovskyy O De Luise M de Biase D Caporali L Fiorini C Gasparre G. Papillary thyroid carcinoma tall cell variant shares accumulation of mitochondria, mitochondrial DNA mutations, and loss of oxidative phosphorylation complex I integrity with oncocytic tumors. J Pathol Clin Res (2022) 8:155–68. doi: 10.1002/cjp2.247 PMC8822387 34792302

[63] Liberzon A Birger C Thorvaldsdóttir H Ghandi M Mesirov JP Tamayo P. The Molecular Signatures Database (MSigDB) hallmark gene set collection. Cell Syst (2015) 1:417–25. doi: 10.1016/j.cels.2015.12.004 PMC4707969 26771021

[64] Hanahan D Weinberg RA. Hallmarks of cancer: the next generation. Cell (2011) 144:646–74. doi: 10.1016/j.cell.2011.02.013 21376230

[65] Stosic A Fuligni F Anderson ND Davidson S de Borja R Acker M. Diverse oncogenic fusions and distinct gene expression patterns define the genomic landscape of pediatric papillary thyroid carcinoma. Cancer Res (2021) 81:5625–37. doi: 10.1158/0008-5472.CAN-21-0761 34535459

[66] Pugh TJ Yu W Yang J Field AL Ambrogio L Carter SL. Exome sequencing of pleuropulmonary blastoma reveals frequent biallelic loss of TP53 and two hits in DICER1 resulting in retention of 5p-derived miRNA hairpin loop sequences. Oncogene (2014) 33:5295–302. doi: 10.1038/onc.2014.150 PMC4224628 24909177

[67] Poma AM Condello V Denaro M Torregrossa L Elisei R Vitti P. DICER1 somatic mutations strongly impair miRNA processing even in benign thyroid lesions. Oncotarget (2019) 10:1785–97. doi: 10.18632/oncotarget.26639 PMC6442996 30956758

[68] Ricarte-Filho JC Casado-Medrano V Reichenberger E Spangler Z Scheerer M Isaza A. DICER1 RNase IIIb domain mutations trigger widespread miRNA dysregulation and MAPK activation in pediatric thyroid cancer. Front Endocrinol (2023) 14:1083382. doi: 10.3389/fendo.2023.1083382 PMC9990750 36896180

[69] Nikiforova MN Mercurio S Wald AI Barbi de Moura M Callenberg K Santana-Santos L. Analytical performance of the ThyroSeq v3 genomic classifier for cancer diagnosis in thyroid nodules. Cancer (2018) 124:1682–90. doi: 10.1002/cncr.31245 PMC5891361 29345728

[70] Schultz KAP Williams GM Kamihara J Stewart DR Harris AK Bauer AJ. DICER1 and associated conditions: identification of at-risk individuals and recommended surveillance strategies. Clin Cancer Res (2018) 24:2251–61. doi: 10.1158/1078-0432.CCR-17-3089 PMC6260592 29343557

[71] Chen J Yang H Teo ASM Amer LB Sherbaf FG Tan CQ. Genomic landscape of lung adenocarcinoma in East Asians. Nat Genet (2020) 52:177–86. doi: 10.1038/s41588-019-0569-6 32015526

[72] Jantrapirom S Piccolo LL Pruksakorn D Potikanond S Nimlamool W. Ubiquilin networking in cancers. Cancers (2020) 12:1586. doi: 10.3390/cancers12061586 32549375 PMC7352256

[73] Jackson JT Mulazzani E Nutt SL Masters SL. The role of PLCγ2 in immunological disorders, cancer, and neurodegeneration. J Biol Chem (2021) 297:100905. doi: 10.1016/j.jbc.2021.100905 34157287 PMC8318911

[74] Moore A Bar Y Maurice-Dror C Finkel I Goldvaser H Dudnik E. Next-generation sequencing in thyroid cancers: do targetable alterations lead to a therapeutic advantage?: A multicenter experience. Medicine (2021) 100:e26388. doi: 10.1097/MD.0000000000026388 34160418 PMC8238320

[75] Nalepa G Clapp DW. Fanconi anaemia and cancer: an intricate relationship. Nat Rev Cancer (2018) 18:168–85. doi: 10.1038/nrc.2017.116 29376519

[76] Bakker ST van de Vrugt HJ Visser JA Delzenne-Goette E van der Wal A Berns MAD. Fancf-deficient mice are prone to develop ovarian tumours. J Pathol (2012) 226:28–39. doi: 10.1002/path.2992 21915857

