# Supplementary material for: Genomic and transcriptomic analyses of thyroid cancers identify DICER1 somatic mutations in adult follicular-patterned RAS-like tumors
Source: Front Endocrinol (Lausanne). 2023 Oct 5;14:1267499. doi: 10.3389/fendo.2023.1267499 (PMC10585144; doi:10.3389/fendo.2023.1267499)
Supplement: Supplementary file 1 [file DataSheet_1.pdf]

Supplementary Material

Genomic and transcriptomic analyses of thyroid cancers identify DICER1 somatic mutations in adult follicular-patterned RAS-like tumors

Minna Emanuela[1*], Devecchi Andrea[1], Pistore Federico[2], Paolini Biagio[3], Mauro Giuseppe[2,4], Penso Donata Alda[1], Pagliardini Sonia[2], Busico Adele[1], Pruneri Giancarlo[5], De Cecco Loris[2], Borrello Maria Grazia[2], Sensi Maria Luisa[6§], Greco Angela[2§*]

*** Correspondence:**
Greco Angela
Angela.Greco@istitutotumori.mi.it

Minna Emanuela
Emanuela.Minna@istitutotumori.mi.it

1 **Supplementary Figure 1.**

2 **Supplementary Figure 2.**

3 **Supplementary Figure 3.**

4 **Supplementary Table 1.**

5 **Supplementary Table 2.** Uploaded as separate excel format table

6 **Supplementary Table 3.**

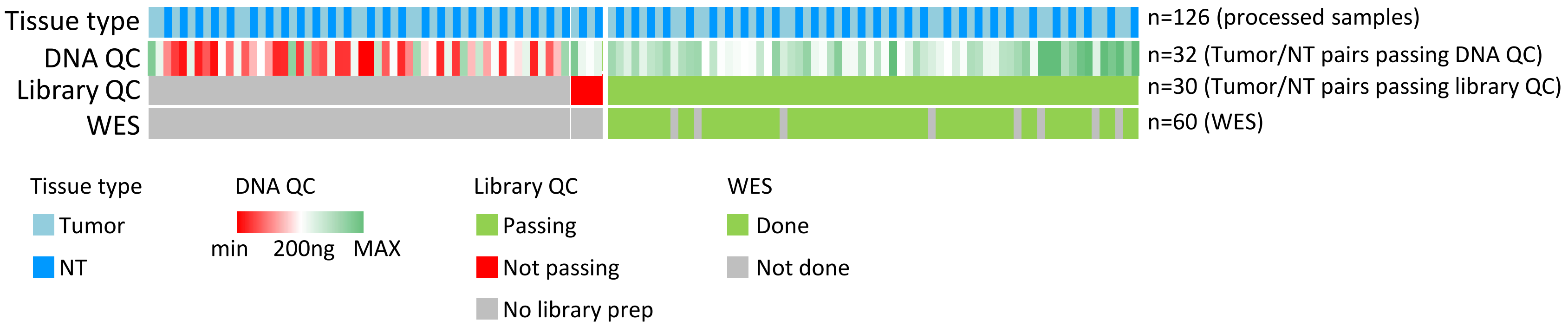

Supplementary Figure 1. Summary of sample processing and selection for whole exome sequencing.
Color code heatmap showing sample processing and quality control; each column represents a tissue sample. A total of 126 tissue sample derived from 57 patients, and including thyroid tumor and patient matched non-neoplastic thyroid, underwent DNA extraction. For 12 patients 2 different tumor specimens were available and processed. Only tumor/NT pairs with both specimens passing DNA quality control (total extracted DNA>200ng) underwent DNA library preparation. Only libraries passing quality control were then submitted to DNA sequencing. For patients with double tumor specimens only the best quality library was sequenced.
Abbreviations: WES, whole exome sequencing; NT, non-neoplastic thyroid; QC, quality control

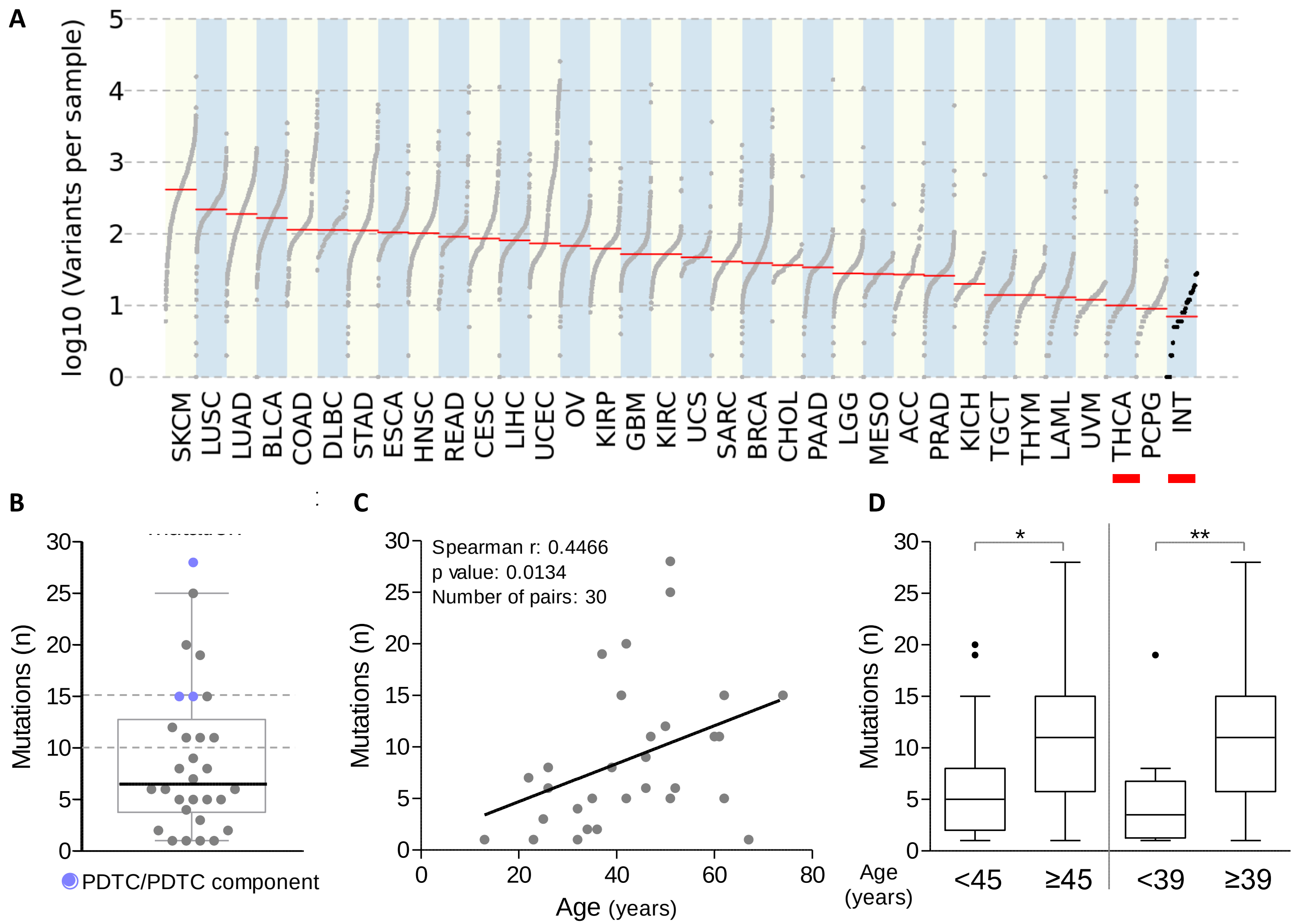

Supplementary Figure 2. Mutation load in the 30 thyroid cancer (TC) patients collected at our Institute.
A. Mutational load distribution in the TC series collected at Fondazione IRCCS Istituto Nazionale dei Tumori (INT) compared to 33 TCGA landmark cohorts. Only non-synonymous mutations (comprising missense, nonsense, and splice-site mutations with amino acid changing) were considered. Each dot corresponds to a tumor sample, with vertical position representing the number of somatic mutations (log10 scale). Cohorts are ordered based on decreasing median of somatic mutation number. Our (INT) and TCGA thyroid cancer (THCA) cohorts displaying similar and low mutational load are highlighted by red solid lines; other tumor type acronyms are described on http://cancergenome.nih.gov/. **B.** Boxplot with scatter plot showing mutational load data (linear scale) in our TC series. **C**. Correlation of mutational load data with patient age in our TC series. **D.** Mutational load data in our TC series stratified for age classes; the 45 year threshold (used in staging systems) and the 39 year threshold (used in young adults definition) were considered. * pvalue<0.05; ** pvalue<0.005 by non parametric Mann Whitney test.

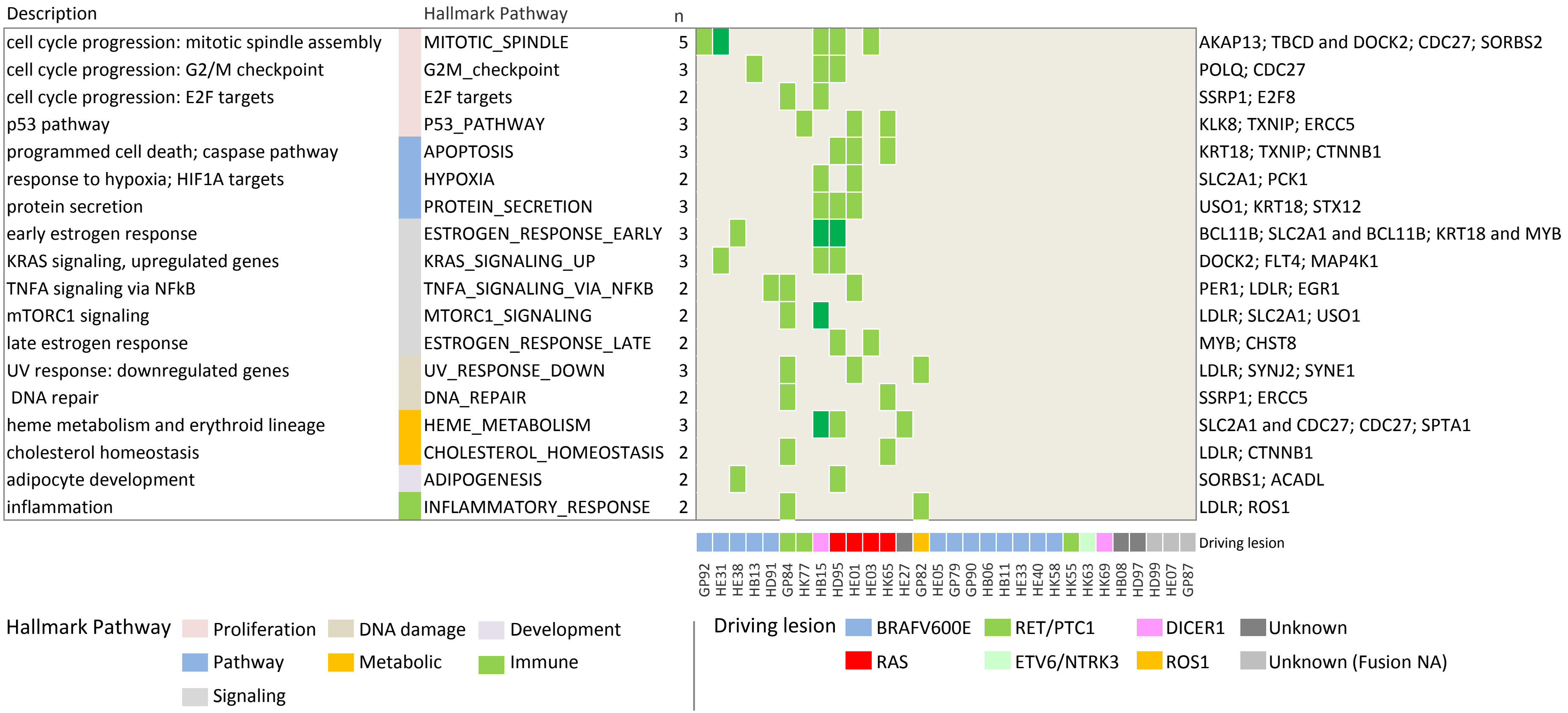

Supplementary Figure 3. Pathway level analysis for somatic mutations identified in our cohort.
Somatic mutations identified in our 30 thyroid cancer patients were investigated at the pathway level to identify the affected biological processes. The Hallmark collection comprising 50 gene sets derived from Molecular Signature Database was tested. A pathway was called mutated if at least two of its member genes were mutated. The identified pathways, the number of mutated samples (n) and the list of mutated genes are showed. Each column represents a patient and the presence of a mutated gene in a given pathway is indicated by the green tag; dark green indicates the concurrence of two mutated genes in the same pathway. Patient ID is shown on the bottom along with driving lesion information. Hallmark pathway color labels indicate biological super categories according to the “Hallmarks” collection original publication.

Supplementary Table 1. Caselist clinicopathological features

Patient ID	Tumor type	Histological subtype	Size (cm)	ETE	LNM (positive/tested)	Age	Gender	Driver summary
HE05	PTC	Tall cell	1.7	Yes	Yes (4/5)	46	F	BRAF_V600E
HE31	PTC	Tall cell	2	Yes	No	50	F	BRAF_V600E
HE38	PTC	Tall cell	1.2	Yes	No	42	F	BRAF_V600E
GP90	PTC	Tall cell	2.2	Yes	Yes (3/4)	61	F	BRAF_V600E
GP92	PTC	Tall cell	1.5	Yes	Yes (6/NA)	32	F	BRAF_V600E
HB11	PTC	Tall cell	5	Yes	No	51	F	BRAF_V600E
HE40	PTC	Tall cell	2.1	Yes	No	62	F	BRAF_V600E
GP79	PTC	Tall cell - trabecular	2.5	Yes	Yes (7/45)	35	F	BRAF_V600E
HE33	PTC	Classical	3.7	Yes	Yes (18/NA)	26	M	BRAF_V600E
HK58	PTC	Classical	1.4	NA	Yes (2/29)	52	M	BRAF_V600E
HB13	PTC	Follicular - classical	1.5	No	No	62	F	BRAF_V600E
HD91	PTC	Follicular	3.4	No	Yes (1/6)	42	F	BRAF_V600E
HB06	PTC	Follicular	1.3	No	No	36	F	BRAF_V600E
HE03	PTC	Follicular	1.8	No	No	22	F	NRAS_Q61R
HE01	PTC	Follicular	1.8	Yes	No	46	F	NRAS_Q61R
HD95	PTC	Follicular	4.5	No	No	37	F	NRAS_Q61R
HK65	PDTC+PTC	NA	2.5	Yes	No	74	F	KRAS_G12V; CTNNB1_Q177*
GP82	PTC	Solid - trabecular	2.8	Yes	No	60	F	ROS1_P2130A
HK69	PTC	Follicular	1.5	No	No	39	F	DICER1_D1810V; DICER1_R459*
HB15	PDTC	Solid - Insular	1.8	NA	No	51	F	DICER1_D1709N
HK55	PTC	Classical	2	No	Yes (25/73)	25	M	RET/PTC1
GP84	PTC	Classical	2	Yes	Yes (4/7)	47	F	RET/PTC1
HK77	PTC	Classical	1.7	Yes	No	13	M	RET/PTC1
HK63	PTC	Follicular with focal oncocytic features	1.8	No	No	34	F	ETV6/NTRK3
HB08	PTC	Classical	1.5	Yes	Yes (3/NA)	32	F	Unknown
HD97	PTC	Follicular	5	Yes	No	51	F	Unknown
HE27	PDTC+PTC	Insular - trabecular with focal PTC	6.5	No	No	41	M	Unknown
HD99	PTC	Follicular	5	No	No	26	M	Unknown (fusion NA)
HE07	PTC	Follicular	4.5	No	No	67	F	Unknown (fusion NA)
GP87	PTC	Solid - trabecular	5	Yes	Yes (17/NA)	23	F	Unknown (fusion NA)

Abbreviations: ETE, extra thyroid extension; LNM, lymph node metastases; PTC papillary thyroid carcinoma; PDTC poorly differentiated thyroid carcinoma; NA, not available

Supplementary Table 3. COSMIC derived thyroid studies reporting ROS1 mutation

Study ID	Mutations (n)	Patients (n)	Total reported samples (n)	Reference PMID	COSMIC ID	ROS1 CDS Mutation	ROS1 AA mutation	ROS1 VAF	TC histotype	Other mutated genes
Pozdeyev_CCR2018	2	2	630	29615459	2762841	c.578T>C	p.V193A	NA	FTC	HRAS_Q61R; BCORL1; RANBP2
					2762794	c.634G>A	p.E212K	NA	PTC	BRAF_V600E; ATM; MEN1; TERT promoter
Swierniak_MCE2016	1	1	48	27283500	2800085	c.4322-10C>A	p. not specified	0.38	FTA	NSD1
Landa_JCI2016	1	1	117	26878173	2666535	c.3626T>A	p.L1209*	0.25	ATC	NF1; PIK3CD; PTEN; TP53 + 23 other genes with SNVs
TCGA_Cell2014*	3	3	402	25417114	2121945	c.3403G>T	p.G1135W	0.12	PTC	BRAF_V600E
					2121996	c.3059C>A	p.P1020H	0.34	PTC	BRAF_V600E
					2121935	c.3239C>A	p.T1080K	0.32	PTC	BRAF_V600E; ZFHX3_H1571R
CGP Study_589	5	4	5	NA	2746518	c.3791G>C	p.R1264T	NA	NA	NA
					2549946	c.3791G>C	p.R1264T	NA	NA	NA
					2186173**	c.2376G>T;	p.M792I;	NA	NA	NA
						c.2983A>T	p.S995C	NA	NA	NA
					2186085	c.6619A>T	p.T2207S	NA	NA	NA
CGP Study_676*	13	10	13	NA	2745043	c.2223C>A	p.H741Q	NA	NA	NA
					2745048**	c.2809C>T	p.P937S	NA	NA	NA
						c.6347G>A	p.R2116K	NA	NA	NA
					2745038	c.5525G>T	p.S1842I	NA	NA	NA
					2745060	c.4561C>T	p.Q1521*	NA	NA	NA
					2745058	c.3833T>C	p.F1278S	NA	NA	NA
					2745049	c.3713T>A	p.L1238H	NA	NA	NA
					2745056	c.2762C>A	p.A921D	NA	NA	NA
					2745055	c.2269G>A	p.G757S	NA	NA	NA
					2745053**	c.2159G>T	p.S720I	NA	NA	NA
						c.2186G>T	p.W729L	NA	NA	NA
						c.2198G>T	p.G733V	NA	NA	NA
					2745059	c.536A>G	p.Y179C	NA	NA	NA
Total	25	21	1215							

* Manually curated (see Material and methods); **ROS1 multiple hits

Abbreviation: CDS, coding sequence; AA, amino acid; VAF, varinat allele frequency (derived from the original publication and cBioportal); TC, thyroid cancer; FTC, follicular TC; PTC; papillary TC; FTA, follicular thyroid adenoma; ATC, anaplastic TC; NA, not available; PMID, PubMed ID
